# Word meaning acquisition is reflected in brain potentials of isolated words

**DOI:** 10.1038/srep43341

**Published:** 2017-03-03

**Authors:** Jan Rouke Kuipers, Anastasia Uminski, Zoe Green, David Hughes, Tommaso Aglietti

**Affiliations:** 1 Psychology Division, Faculty of Natural Sciences, Stirling University, FK9 4LA Stirling, United Kingdom

## Abstract

Learning a new concept and corresponding word typically involves repeated exposure to the word in the same or a similar context until the link crystallizes in long term memory. Although electrophysiological indices of the result of learning are well documented, there is currently no measure of the process of conceptually-mediated learning itself. Here, we recorded event-related brain potentials from participants who read unfamiliar words presented in isolation followed by a definition that either explained the meaning of the word or was a true, but uninformative statement. Self-reported word knowledge ratings increased for those words that were followed by meaningful definitions and were correlated with a decrease in ERP amplitude of a late frontal negativity (LFN) elicited by the isolated word. Importantly, the rate of LFN amplitude change predicted post-hoc learning outcome measures. Therefore, the LFN is real-time measure that is not under conscious control and which reflects conceptually-mediated learning. We propose that the LFN provides for the first time the opportunity to assess learning during study.

Humans have the capacity to learn a vast amount of words in their lifetime with estimates of adult vocabularies ranging from 40,000 to 90,000 words^1^,^2^. Word learning can be achieved by fast mapping novel words onto linguistic and semantic information provided by the context^3^,^4^ which is assumed to be a general learning strategy also observable in non-human animals^5^. Conceptually-mediated learning -here defined as modifying existing concepts or creating new ones and linking them to novel word forms- has been shown to involve the left-prefrontal cortex^6^ and anterior temporal lobes^7^ whilst the extent of hippocampal involvement in this process is debated^8^,^9^.

Learning in adults is frequently studied from the perspective of second language (L2) learning^10^,^11^,^12^ or pseudoword learning^13^. Such word learning typically involves mapping novel word forms to first language (L1) words eventually leading to a single concept represented by two words^14^. Therefore, L2 word and pseudoword learning may not reflect learning a novel concept and a corresponding word, because the concept the novel word form refers to is already established and mapped onto an existing L1 word. Furthermore, studies of (word) learning typically involve measuring explicit (conscious button presses) or implicit (unconscious, electrophysiological) responses after a learning phase which affords limited insight in the learning process itself. Although attention allocation during learning can be predictive of later recollection^12^,^15^, there is currently no implicit measure of the process of conceptually-mediated learning.

Here, we aimed to characterize event-related brain potential (ERP) correlates of conceptually-mediated word learning in a naturalistic learning setting. ERPs are scalp-recorded fluctuations in electrical activity that represent systematic neural responses related to stimulus processing which provides the opportunity to study cognitive processes (e.g., meaning integration) with high-temporal resolution. We selected words from online scientific glossaries that undergraduate Psychology students are unlikely to know (e.g., from endocrinology). These words were presented in isolation followed by a different statement about their meaning each time with a total of ten presentations. Only half of the words were followed by meaningful statements (the learn condition; e.g., “Vasopressin is a hormone that alters kidney functioning”), thus knowledge about these words only was assumed to increase incrementally. The words in the fill condition were followed by true, but non-informative statements, leading an increase in their familiarity whilst knowledge about the meaning of these words was not increasing (e.g., “Vasopressin has been found in prairie voles”). During this learning phase, participants were instructed to indicate with a button press how well they knew the meaning of the target word after they had read the sentence (Fig. 1). These knowledge ratings were expected to change with increasing number of definitions in the learn condition only.

Given that the N400 ERP component is a sensitive index of meaning processing^16^, our priory assumption was that the extent to which a semantically isolated word can be mapped onto a stable concept should be reflected in its N400 amplitude. Easier meaning integration typically results in more positive N400 amplitudes^16^ and therefore we anticipated that a gradual increase in stability of a concept and its link with a word form would be reflected in a gradually increasing positivity of the N400. However, given that stimulus repetitions also elicit more positive N400 amplitudes^17^, we anticipated to find a modulation of N400 amplitude by the number of repetitions as well as a modulation by increasing word knowledge.

To assess whether participants had learned the meanings of the words in the study phase, we presented all words from the learn and fill conditions at the end of true and false sentences in a test phase. These sentences, in which we avoided using key words used in the definitions, were designed to test word knowledge (e.g., “When you are thirsty your body produces vasopressin”). Participants indicated correctness of these sentences with a button press while we recorded ERPs elicited by the sentence-final test words. We expected to find a typical N400 semantic priming effect in the learn condition only, paired with shorter reaction times and fewer errors for words from the learn condition as compared to the fill condition.

## Results

### Behavioral responses during learning

We analyzed self-rated word knowledge responses using linear mixed effects modelling with repetition (1–10) and condition (learn, filler) as fixed variables, and participants and items as random variables. We found significant effects of Condition (F_(1,2831)_ = 119, p < 0.001), Repetition (F_(9,2828)_ = 7.1, p < 0.001), and their interaction (F_(9,2831)_ = 2.5, p < 0.01). Follow-up analyses on each condition separately revealed that the effect of repetition was significant in the learn condition (F_(9,1398)_ = 8.1, p < 0.001) but not in the fill condition (F_(9,1398)_ = 1.6, p = 0.1) confirming that word knowledge ratings increased in the learn condition only (Fig. 2).

### Brain responses during learning

Next, to test whether ERPs also changed while participants were learning, we first visually inspected grand average ERP waveforms of all word repetitions in each condition. The total of ten item presentations were collapsed using a sliding average over four subsequent repetitions in order to obtain a sufficient number of trials in each individual’s ERP average (32 trials for each set of collapsed repetitions: 1–4, 2–5, …, 7–10). The repetition and learn conditions visually modulated P2 amplitude at frontal electrode sites (Fig. 3) and N400 amplitude at centro-parietal electrodes (Fig. 4). Unexpectedly, repetition effects were also visible at frontal electrodes from 700 to 900 ms after stimulus onset (Fig. 3). Each of these modulations by stimulus repetition and learning context were analyzed with analyses of variance (ANOVAs).

Mean P2 amplitude was modulated by Repetition (F_(2.7,49.2)_ = 5.3, p = 0.004) but not in the same way in each learning condition, as shown by a significant interaction between repetition and learning condition (F_(2.5,44.6)_ = 3.1, p = 0.046). Follow-up regression analyses –chosen to avoid multiple t-tests due to the many levels of the factor repetition- revealed that the P2 incrementally increased in amplitude with each repetition in the fill condition (B = 0.21, t = 2.5, p = 0.011). However, P2 amplitude did not change with increasing repetitions in the learn condition (B = 0.09, t = 1.3, p = 0.199). Therefore, when the familiarity of a word increases but its meaning remains unknown, the attention-related P2 brain response^18^ increases in amplitude, whereas this is not the case when word knowledge and familiarity both increase with increasing item repetitions.

Next, the N400 became more positive with increasing word repetitions (F_(2,37)_ = 7.3, p = 0.002; Fig. 4) while it was neither modulated by learning condition (p = 0.22) nor the interaction between repetition and learning (p = 0.82). Therefore, since the N400 amplitude is similarly modulated by word repetition whether or not participants are learning, the N400 does not appear to be a reliable index of conceptually-mediated learning.

Given that the late-frontal negativity (LFN) was an unanticipated, novel effect, we analyzed it over the whole scalp using frontal, medial and parietal-occipital clusters of electrodes. This analysis showed that LFN modulations differed between scalp regions (F_(1.2,21.2)_ = 22.7, p < 0.001) and the modulation by learning condition differed between scalp regions as reflected in a significant three-way interaction between region, learning condition, and repetition (F_(3.1,57)_ = 4.2, p = 0.008). Separate analyses for each region revealed that the effect of condition was not significant in any region (all p > 0.07) and that the LFN modulation was not significant in the central and the parietal-occipital regions (both p > 0.07). However, in the frontal region the interaction between repetition and learning condition was significant (F_(2.7,49.2)_ = 5.9, p = 0.002). Follow-up regression analyses revealed that LFN linearly increased in negativity over the different repetitions in the learn condition (B = −0.23, t = 2.4, p = 0.019), but did not significantly change in the fill condition (B = 0.07, t = 0.8, p = 0.399). Therefore, LFN amplitude decrease seems to reflect conceptually-mediated word learning. Below we test the reliability and predictive value of the LFN as a marker of conceptually-mediated learning.

### Linking brain and behavioral responses during learning

To test whether participants’ word knowledge ratings are associated with their electrophysiological brain responses we performed correlation analyses between significant effects of the two measures. These revealed that in the learn condition, as knowledge ratings increased, N400 amplitude became more positive (R = 0.65, p = 0.04), whereas the LFN became more negative in amplitude with increasing knowledge ratings (R = −0.71, p = 0.02). The N400 repetition values did not correlate with those of the LFN (p = 0.3) showing that the repetition effects of these components were functionally independent.

In the fill condition, self-reported learning ratings did not increase; therefore no correlations were performed on these measures. However, given that the P2 and N400 were significantly modulated by repetition in this condition, we did perform a correlation analysis revealing that an increase in P2 amplitude was associated with a more positive N400 (reduction in its amplitude; R.76, p = 0.01). This suggests that that P2 and N400 modulations by different repetitions may be functionally linked.

### Behavioral responses after learning

To test whether the above effects are due to conceptually-mediated learning we analyzed behavioral and N400 responses in the test phase. The words of the learn and fill conditions were this time presented in a semantic context of true and false sentences. Significant effects of validity and learning condition showed that mean reaction times (RTs) to false sentences (1030 ms) were faster than to true sentences (1163 ms; F_(1,17)_ = 11.1, p = 0.004), and that learn sentences (1055 ms) were responded to faster than filler sentences (1138 ms; F_(1,17)_ = 8.3, p = 0.01). In the error analysis, following significant effects of truth (F_(1,17)_ = 16.7, p = 0.001), condition (F_(1,17)_ = 8.4, p = 0.01), as well their interaction (F_(1,17)_ = 12.9, p = 0.002), post-hoc t-tests revealed that true items were rejected more often in the fill (28% of trials) than learn condition (18%; p = 0.003), whereas false items were accepted equally often (both 10%; p = 0.570). D-prime for the learn condition was d′ = 1.3 while d′ = 0.7 was observed in the fill condition. These behavioral results confirm that participants had learned more from words followed by meaningful definitions than filler conditions because they responded faster and more accurately to items previously presented in the learn condition.

### ERP responses after learning

Inspection of the grand average ERPs of the test phase revealed the predicted N400 difference between true and false sentences observed in the learn condition only (Fig. 5). The ANOVA on mean N400 amplitude (350–450 ms) revealed significant effects of truth (F_(1,18)_ = 17.2, p = 0.001) and the interaction between truth and condition (F_(1,18)_ = 9.9, p = 0.006). Follow-up analyses showed that the N400 effect (3.4 μV) was significant in the learn condition (p < 0.001) whilst absent in the fill condition (0.01 μV; p = 0.98).

### LFN amplitude as an index of conceptually-mediated learning

Finally, we investigated whether the incremental decrease in LFN over the different repetitions can be used as an online index of how much conceptual word learning is taking place. We first calculated the regression slope for each item (both fill and learn conditions) and each participant over the different repetitions for both the LFN and N400. These slopes indicate whether over the different repetitions, an item for a participant increased or decreased in N400 and LFN amplitude. LFN slope differed between learn and fill conditions (t_246_ = 2.05, p = 0.042) whereas N400 slopes did not (t_246_ = 0.02, p = 0.46; Fig. 6). Next, given that the average N400 amplitude of the test phase is indicative of whether an item had been learned, we used the trial-by-trial slopes of each item and participant as predictors in a regression on each item’s and participant’s N400 effect of the test phase (i.e. the average amplitude of 5 matching trials minus the average amplitude of 5 mismatching trials). The results show that the slope of LFN decrease significantly predicts N400 amplitude of the test phase (B = 1.24, t = 3.66, p < 0.001) whereas the slope of N400 change does not (p = 0.49). Therefore, given that N400 amplitude in the test phase indexes whether or not any conceptually-mediated learning has taken place previously, a decrease in LFN amplitude over different word repetitions (a negative slope) indexes that conceptually-mediated learning is taking place.

## Discussion

The aim of this study was to identify ERP correlates of conceptually-mediated learning. To create a realistic, conceptually-mediated learning context, we used real words which meanings were unknown to the participants and paired them with a different definition at each presentation. In this learning phase, the only ERP modulation that selectively responded to learned items was a late frontal negativity (LFN). With increasing word knowledge, the LFN elicited by isolated words became gradually more negative and the rate of this decrease reflects how well an item is being learned. The LFN did not correlate with any other ERP measure suggesting this is an independent ERP index of word knowledge.

We further found that P2 amplitude increased in amplitude in response to increasingly familiar but semantically unknown words. The P2 is generally associated with attention allocation^18^ and with lexical-semantic access in word production^19^. Therefore, this result suggests that the attention response at the time of lexical access becomes larger when a word form is increasingly familiar but not linked to any semantic information in long term memory. Conversely, the P2 response did not increase for words in the learn condition suggesting that when the meaning of a word is known and can thus be linked with a semantic representation, the attention allocated during lexical-semantic access remains at a baseline level. We propose that failing to identify a word form in long term memory seems to increase attention paid to this word. Well known words have established links with concepts in long term memory and may not evoke an increased attentional response.

An important aspect that differentiates the current study from previous ones is that we recorded ERPs from isolated words instead of words embedded in a semantic context. In a typical (N400) semantic priming paradigm, the test-word is placed at the end of a sentence following the logic that, when the meaning of a word is known it will be semantically primed by the sentence context, which reduces N400 amplitude it elicits. However, given that in this scenario the N400 of the target word is dependent on its semantic fit with the context, it is impossible to assess the strength of the conceptual representation activated by the target word. Difficulty in integrating a word in its semantic context may be due to a semantic mismatch of the word when it is known, or due to a lack of semantic knowledge of the word. In both cases N400 amplitude becomes more negative. Therefore, to eliminate semantic context effects on ERPs of word processing, we measured ERPs elicited by contextually isolated words, i.e., at the beginning of a sentence.

Contrary to our predictions, N400 amplitude was not modulated by an increase in semantic word knowledge. Instead, the N400 increased in amplitude with increasing repetitions regardless of whether participants were learning about the meaning of the word. Therefore, an N400 elicited by an isolated word does not appear to reflect semantic knowledge of that word but seems to predominantly reflect word familiarity instead. This sensitivity of the N400 to repetition effects has been previously reported in memory studies^17^,^20^ and may therefore confound its potential response to word learning. Nevertheless, the N400 is suitable to measure learning outcome by means of semantic priming, which we used in the test phase of the experiment. Faster and more accurate behavioral responses and a large N400 modulation for words in the learn condition as compared to words in the filler condition confirmed that participants had only learned the meaning of those words that had been followed by meaningful definitions.

The LFN modulation we observed is reminiscent of the difference due to memory effect (DM effect)^21^, which is the larger ERP amplitude elicited by items that are better remembered later on. However, the DM effect is based on item recollection and not on conceptually-mediated learning and was therefore not anticipated. Furthermore, the DM effect is opposite in amplitude of the LFN and maximal over central as compared to frontal electrode sites and is therefore unlikely to reflect the same cognitive process as reflected by the LFN.

Recent neuroimaging studies have revealed an important role of the prefrontal cortex in memory control^22^ and retrieval^23^. Here, we observed that learning the meaning of words modulated frontal electrode sites, but unfortunately, electrode location bares little information about the location of the neural source^24^. However, given that the LFN is sensitive to the fast mapping of meaning and that hippocampal ERP signals are difficult to detect non-invasively^25^, we tentatively propose that the LFN may reflect fast neocortical learning involving the prefrontal cortex^7^,^26^.

Future studies may further assess trial-by-trial sensitivity of the LFN as an online marker of learning by using a more typical learning context. Here, the learning experience was challenging because participants were presented with unknown words of different disciplines they were unfamiliar with. These disciplines changed randomly from trial to trial and words were only followed by a single short definition each time they were presented. In addition, half of the unknown words were followed by uninformative definitions which may have created confusion on part of the participants. By contrast, in a classroom context, students know which topic they learn about and new concepts are explained exhaustively, including repetitions of the same facts before a new concept is introduced. Several studies have shown that learning the meaning of a word is stimulated when the context is informative and contains familiar words related to the word^27^,^28^,^29^.

To conclude, here we report a late frontal negativity (LFN) which is a novel ERP that is selectively modulated when participants learn the meaning of unfamiliar words. LFN amplitude during learning correlates with self-reported learning ratings suggesting that the LFN as an implicit record of brain activity (i.e. not being under conscious control) corresponds with a later, conscious judgment of word knowledge. Importantly, the rate of LFN amplitude change during word learning predicts N400 amplitude at test where it was used as a learning outcome measure. These observations strongly suggest that the LFN of an isolated word uniquely indexes how well the meaning of that word is known. However, it is still an open question whether without an adequate baseline, LFN amplitude of a single word after one exposure indexes knowledge of that word. Instead, insight in word knowledge seems reflected in the amount of LFN change over different word repetitions. Our results show that the extent to which someone learns new information about a word is reflected in the change of the LFN elicited by that word, provided the word is presented outside a direct semantic context. Therefore, we propose that an increase in negativity of the LFN reflects growth and stabilization of the conceptual representation linked to a word form. The LFN provides for the first time the opportunity to establish online, without conscious report on part of the participant, whether the participant is learning new information or capable of learning in, for example, a clinical context.

## Methods

### Participants

Twenty-one undergraduate students participated in exchange for course credits. Participants were native English speakers with normal or corrected-to-normal vision, none reported being dyslexic. One participant was left-handed as assessed by the Edinburgh Handedness Inventory^30^. The experiment was performed in accordance with relevant guidelines and regulations and approved by the ethics committee of Stirling University. Written consent was obtained from all participants. Two participants were excluded due to an insufficient number of artefact-free trials (<18 in the test phase). The remaining participants’ age was 20 +/− 1.3 years and 12 were female. The behavioral data of the test phase of one additional participant were not correctly recorded and therefore not included in the behavioral analysis.

### Stimuli

Sixteen words (8–13 letters in length), four from each discipline of Astronomy, Endocrinology, Pharmacology, and Physics, were selected from scientific glossaries if their meaning was anticipated to be unknown to Psychology undergraduate students. For each of these words, ten definitions were created that provide insight into the meaning of each word (learn definitions; e.g., Vasopressin is a hormone that alters kidney functioning). In addition, ten statements were created for each word that do not bare information about its meaning (fill definitions; e.g., Vasopressin has been found in prairie voles). The critical words were placed at the heads of the sentences (160 in total) in order to record ERPs of these words outside a semantic sentence context.

For the test phase of the experiment, 160 sentences were created that ended with each of the 16 words (10 sentences for each word). Half of the sentences were true and based on the meaning provided in the true definitions, while avoiding using key words used in the definitions (e.g., True sentence: When you are thirsty your body produces vasopressin). These sentences and critical words were re-paired to create false sentences (e.g., “To learn about our galaxy one could use vasopressin”).

### Procedure

Two stimulus lists were created in which for each discipline half of the words were paired with learn definitions whilst the other two words of the discipline were paired with fill statements. This pairing was counterbalanced across stimulus lists. Participants were presented with one list in a randomized order counterbalanced across participants.

**Figure 1:**
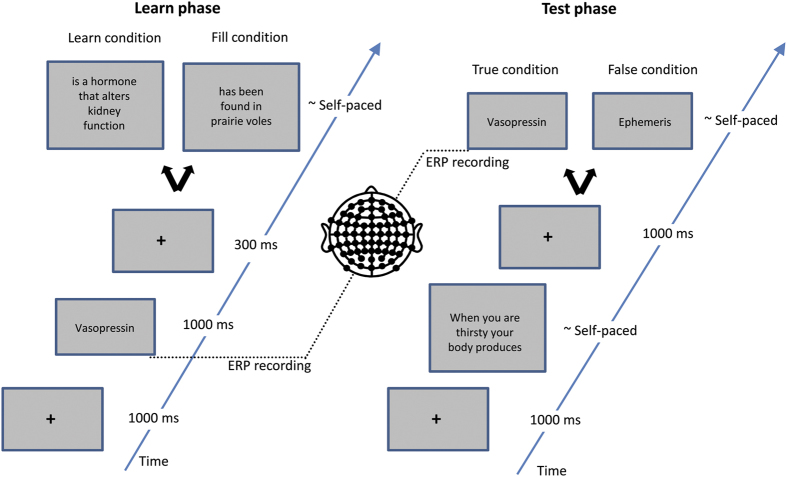
Trial procedure in the learn (left) and test (right) phases.

**Figure 2:**
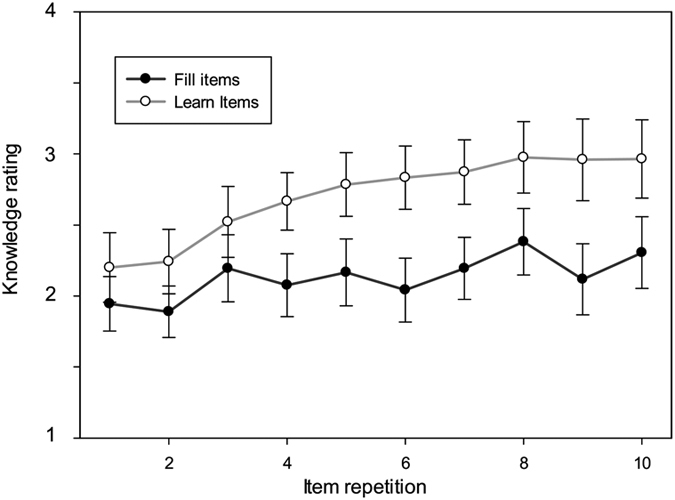
Self-reported word knowledge ratings. The manual word knowledge ratings (score 1: no word knowledge; score 5: knows the word meaning very well) and their standard errors of words in the learn condition (black line) and filler condition (grey line) over the 10 word repetitions.

**Figure 3:**
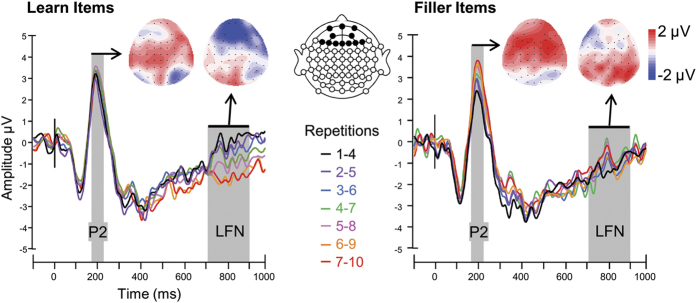
Grand average ERP waveforms of frontal electrodes in the learning phase. A linear derivation of frontal electrodes (Fp1, FP2, FPz, AF3, AF4, F1, F2, F3, F4, F5, F6, Fz) of all word repetitions (1–10, collapsed over 4 repetitions) in the learn and fill conditions. Grey shaded areas indicate the P2 and LFN and the colored scalp outlines the topographies of these effects which were calculated by subtracting the last four repetitions from the first four repetitions. Stimulus onset is indicated by the vertical black line at 0 ms. The epoch is increased from 900 ms to 1000 ms post stimulus onset for illustration purposes only.

**Figure 4:**
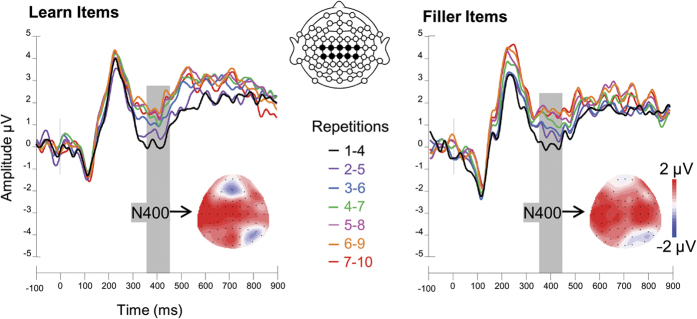
Grand average ERP waveforms of central electrodes in the learning phase. A linear derivation of central-parietal electrodes (C1, C2, C3, C4, Cz, CP1, CP2, CP3, CP4, CPz) of all word repetitions (1–10, collapsed over 4 repetitions) in the learn and fill conditions. Grey shaded areas indicate the N400 and the colored scalp outline its topography which was calculated by subtracting the last four repetitions from the first four repetitions. Stimulus onset is indicated by the vertical black line at 0 ms.

**Figure 5:**
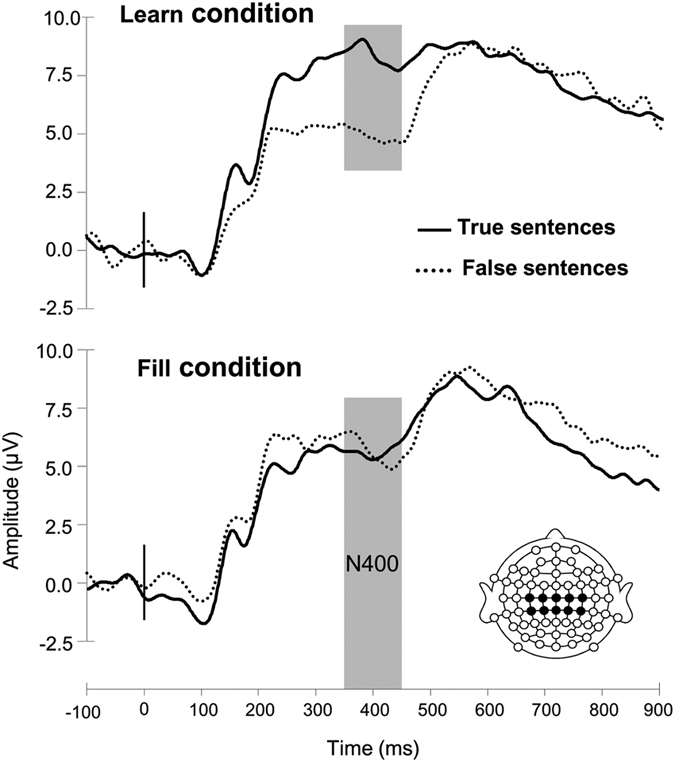
Grand average ERP waveforms of the test phase. A Linear derivation of central electrodes (C1, C2, C3, C4, Cz, CP1, CP2, CP3, CP4, CPz) of true and false sentences for words previously presented in the learn and filler conditions. The N400 is indicated with the grey bar and stimulus onset by the vertical black line at 0 ms.

**Figure 6:**
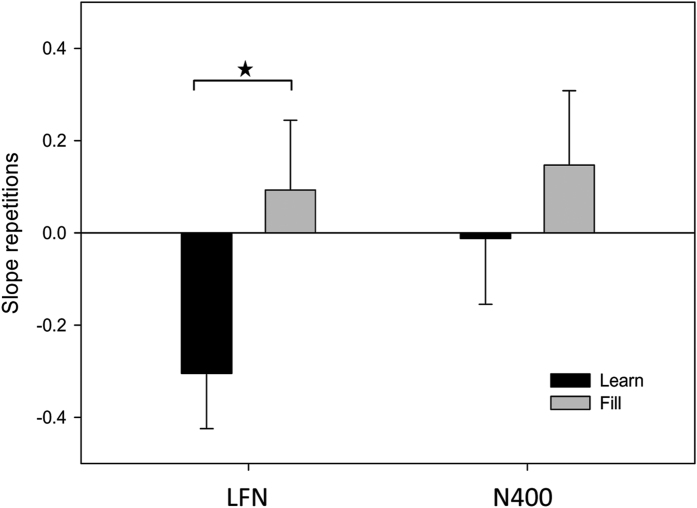
Average LFN and N400 regression slopes. The average regression slopes of 10 repetitions and their standard errors of the LFN and N400 in the learn (black bars) and fill (grey) conditions. The star indicates a statistically significant difference at p < 0.05.

The experiment was organized in a learn phase and a test phase each starting with two practice trials. Each trial in the learning phase began with presentation of a fixation cross presented for 1 s, replaced by the target word (duration 1 s), and finally the remainder of the sentence until the participant pressed a button on a response box to indicate how well they knew the meaning of the word using a 1–5 Likert scale (score 1 = “I do not know the meaning of the word at all”; score 5 = “I know the meaning of the word very well”; Fig. 1).

In the test phase, each trial began with a fixation cross in the middle of the screen for 1 s, followed by a prime sentence terminated by a button press when the participant finished reading the sentence, a 1 s inter stimulus interval with a central fixation cross followed by the target word until the participants’ true/false button press. In total, participants completed 320 trials in 5–6 min blocks interspaced by short breaks.

### Event related potentials

Electrophysiological data were recorded using a Synamps II amplifier (www.neuroscan.com) connected to a 64-channel Ag/Ag/Cl Quik-Cap (NeuroMedical Supplies). Electrodes on the cap were arranged according to the extended version of the 10–20 system. Vertical and horizontal eye movements were monitored using pairs of electrodes placed on the outer canthi of both eyes and one above and one below the left eye. Two additional electrodes were placed on each mastoid bone. Data was recorded at a rate of 1 kHz, filtered online with a band pass between 0.01 Hz and 200 Hz, using a reference electrode located between Cz and CPz and one between Fz and FPz as ground. Impedance of the electrodes was kept below 5 KOhm.

Raw EEG data were filtered offline using a zero phase-shift band pass filter of 0.1 Hz (12 db/Oct) to 30 Hz (48 db/Oct). The continuous data were re-referenced to the average of the mastoid electrodes, mathematically corrected for eye blinks, visually inspected, and epoched from −100 to 900 ms relative to target word onset. Epochs were baseline corrected over the pre-stimulus interval and rejected when any cap electrode exceeded +/−75 μV.

To obtain a sufficient number of trials in each individual ERP average in the learn phase, we applied a sliding average over 4 repetitions (1–4; 2–5, etc.; resulting in 29 +/− 2.3 trials on average for each cluster of repetitions) of each condition. Individual averages of the test phase included 31 +/− 3 trials on average per condition.

### Analyses learn phase

RTs of the self-reported knowledge ratings were not analyzed because they reflect sentence reading times and sentences were slightly longer in the learn condition (8.3 words on average) than fill condition (5.6 words on average; p < 0.001).

Mean P2 amplitudes were calculated from 170–230 ms relative to word onset over all frontal electrodes which is where the P2 modulation was maximal and typically observed (Fp1, FP2, FPz, AF3, AF4, F1, F2, F3, F4, F5, F6, Fz; ref. 18). Mean N400 amplitudes were analyzed using the typical N400 time-window and electrodes (350–450 ms at C1, C2, C3, C4, Cz, CP1, CP2, CP3, CP4, CPz; ref. 16). Since the late-frontal effect was neither anticipated nor resembling any previously documented ERP component, it was analyzed over the whole scalp using the mean amplitude from 700–900 ms. All ERP components were analyzed with repeated measures ANOVAs with Repetition (collapsed repetitions 1–4, 2–5, 3–6, 4–7, 5–8, 6–9, 7–10) and Type of definitions (meaningful vs meaningless) as within subjects variables except for the analysis on the late-frontal effect which also included the factor Region (frontal: the same electrodes as used for the P2; central: FC1–6, FCz, C1-6, Cz, CP1-6, CPz; parietal-occipital: P1-6, Pz, PO3-6, O1-2, Oz).

Correlations between significant behavioral and ERP effects were performed on individual averages to investigate potential relationships between behavioral and ERP measures as well as between the different ERP measures.

### Analyses test Phase

RTs and error rates in response to the true and false sentences were subjected to an ANOVA with Truth (true vs. false sentences) and Condition (Learn vs. fill) as within subjects factors. RTs below 300 ms (3 trials) and above 3 s (153 trials) were excluded from analysis.

Mean N400 effects were calculated as above and subjected to an ANOVA with Condition (Learn vs. fill) as a within subjects factor.

Greenhouse-Geisser and Bonferroni corrected values are reported where applicable.

## Additional Information

**How to cite this article:** Kuipers, J. R. *et al*. Word meaning acquisition is reflected in brain potentials of isolated words. *Sci. Rep.* **7**, 43341; doi: 10.1038/srep43341 (2017).

**Publisher's note:** Springer Nature remains neutral with regard to jurisdictional claims in published maps and institutional affiliations.
